# Are Happy Workers More Productive? The Mediating Role of Service-Skill Use

**DOI:** 10.3389/fpsyg.2020.00456

**Published:** 2020-03-27

**Authors:** Andrés Salas-Vallina, Manoli Pozo-Hidalgo, Pedro R. Gil-Monte

**Affiliations:** ^1^Deparment of Business Management, University of Valencia, Valencia, Spain; ^2^Department of Social Psychology, University of Valencia, Valencia, Spain

**Keywords:** happiness at work, service-skill use, performance, affective organizational commitment, job satisfaction, engagement, cross-selling

## Abstract

The purpose of this paper is to examine the relationship between happiness at work and cross-selling performance in the banking sector. In addition, the mediating effect of service-skill use is analyzed in the relationship between happiness at work and performance. Confirmatory factor analysis is used by means of structural equation models to assess the relationship between happiness at work, service-skill use, and cross-selling performance. A sample of 492 financial service employees is examined. Results reveal that happiness at work positively and directly affects cross-selling performance. The study also shows that service-skill use plays a partial mediating role in the relationship between happiness at work and cross-selling performance. This research expands the theory of the happy productive worker perspective based on the job demands-resources model and defines and conceptualizes service-skill use. Employees who are happier at work cross-sell better, but their service-skill use mediates the effect of happiness at work on cross-selling performance.

## Introduction

In the current highly uncertain and competitive business environment, positive attitudes can become a fundamental source of competitive advantage and success (Guest, 2017). Thereby, particular attention has been paid to the antecedents of cross-selling performance (Yu et al., 2018). The effect of emotions on consumers has recently been addressed by Guido et al. (2018), who found a clear relationship between emotions and consumers’ attitudes. Schmitz et al. (2014) found that intrinsic motivation led to an improvement in cross-selling performance in the banking sector. Zeffane et al. (2017) underlined the increasing importance of positive attitudes, such as job satisfaction, in different firm outcomes, following the positive attitudes-performance line of research (Judge et al., 2001). Chadi and Hetschko (2018) highlighted the essential role of job satisfaction in improving performance. A company can push sellers to sell more, but if sellers are not happy at work, they will not be motivated to sell to the best of their ability. Based on the above, the aim of this paper is twofold: (1) to examine the effect of employees’ happiness at work (HAW) on cross-selling performance and (2) to explore the mediating role of service-skill use in the relationship between HAW and cross-selling performance.

In banking services, cross-selling is expected of frontline branch employees. Cross-selling is the action or practice of selling an additional product or service to an existing customer and helps the company to increase profits (Kamakura et al., 2003). It is a common practice in service industries because face-to-face interactions with customers enable the seller to suggest new services they can offer. In banking, for example, it is common to offer several products at once (mortgages, life insurance, credit cards, and pension plans). Cross-selling can considerably increase the sales volume per customer as a result of transforming a single product or service into multiple products or services (Kamakura, 2008). By way of example, Kamakura et al. (2003) found that increasing the number of products a customer uses from three to four doubled the firm’s profitability. Therefore, it is important to determine which factors can increase cross-selling in a company. Cross-selling requires sales skills and product knowledge, which employees may lack (Yu et al., 2018). However, positive attitudes involving personal well-being could mitigate this lack of skills and knowledge. In particular, wide positive attitudes improve positive behaviors (Salas-Vallina et al., 2017a) because they help employees to face complex and challenging situations. A positive working context, where employees are proactive and energetic, positively affects cross-selling (Yu et al., 2018).

Nonetheless, the direct relationship between positive attitudes and performance has not been proved (Iaffaldano and Muchinsky, 1985; Judge et al., 2001). Theoretically, different mechanisms have been put forward to try to explain why happy workers perform better (Bakker and Demerouti, 2008). For example, positive emotions develop individuals’ “thought–action repertoires,” thus increasing personal resources (Fredrickson, 2013). In addition, engaged people are healthier, so they can put more energy into their work. Happier employees also inspire their colleagues, which promotes networking quality and performance. In this study, we use the concept of HAW that was proposed by Fisher (2010), and empirically checked by Salas-Vallina et al. (2017a). HAW is defined as an attitudinal state of engagement, job satisfaction, and affective organizational commitment. It is a wide attitudinal construct with a specific property: it overcomes the compatibility principle, whereby wide positive attitudes explain job behaviors better (Harrison et al., 2006). The job demands–resources (JD-R) model argues that job resources (physical, psychological, social, and organizational aspects) lead to improved behaviors, while job demands result in negative outcomes, such as burnout. In addition, job resources can also reduce job demands and lead to improved organizational behaviors (Demerouti et al., 2001). Accordingly, HAW can act as a powerful psychological job resource resulting in increased cross-selling performance.

However, empirical findings have varied considerably across studies, depending on the conceptualization of the term “happiness.” While Iaffaldano and Muchinsky (1985) showed a poor relationship between job satisfaction and job performance, Judge et al. (2001), in a meta-analysis, found higher correlations, yet they found low values. Both job satisfaction and engagement have shown positive effects on performance (Wright and Cropanzano, 2000; Bakker and Bal, 2010). Accordingly, our first objective is to check whether HAW positively affects cross-selling performance.

Another aspect influencing performance is personal resources (Bakker and Demerouti, 2008). In this study, service-skill use was proposed as a personal resource mediating the relationship between HAW and cross-selling performance. The term *service-skill use* was defined based on Wang and Xu’s (2017) understanding as the level of use of employees’ communication skills, relationship skills, efficiency, and effectiveness toward the customer. Service-skill use is basically performed to foster persuasion and influences people’s relationships and interactions. Service-skill use is essential for performance, as it promotes customer-oriented employees (Manna, 2017). Specifically, this paper states that in companies in which sellers are happy at work, their communication abilities, interpersonal relationships, efficiency, and effectiveness toward the customer are fostered, in turn increasing their cross-selling performance. Employees’ skill use is considered to be an internal contingency factor that affects dynamic capabilities (Gremme and Wohlgemuth, 2017), and communication abilities are considered to be an essential source of performance (Binyamin and Brender-Ilan, 2017). This relationship between service-skill use and cross-selling occurs as a result of the increase in the perceived value of consumers due to better interactions between service providers and customers (Vargo and Lusch, 2004). According to the JD-R model, we can argue that HAW, as a job resource, reinvigorates employees’ motivation, thus improving service-skill use (employees are motivated to exploit their knowledge as well as learn new skills if required), resulting in improved cross-selling performance. Thus, HAW and service-skill use interact, creating a robust potential, which can impact on performance. Hence, our second objective is to assess the mediating role of service-skill use in the relationship between HAW and cross-selling performance. Therefore, the aim of this paper is to expand the research line of positive attitudes in the banking industry (Cegarra-Navarro et al., 2018).

This paper is organized as follows. First, a theoretical review and hypothesis development is presented. Then, methods and results are explained, and a discussion of theoretical and practical implications is put forward. Finally, limitations and future research directions are suggested.

### Conceptual Background and Hypotheses

#### HAW and Cross-Selling Performance

Happiness at work can be considered as an attitude, as it is a way of feeling about something that precedes a behavior (Salas-Vallina et al., 2017a). Happier employees report better outcomes than less happy employees (Wright et al., 2002). Fredrickson (2001) affirmed that positive attitudes (engagement, job satisfaction, HAW) build resources for future performance, predicting long-term productivity. The common point of all these studies is that happier and more satisfied workers will perform better in their jobs.

However, positive attitudes-performance has sometimes provided unexpected results (Iaffaldano and Muchinsky, 1985; Judge et al., 2001). For example, Martin et al. (1993) showed that positive moods result in perseverance when people work until they feel like stopping. In a meta-analysis of 54,417 observations of 312 samples, Judge et al. (2001) found a correlation between job satisfaction and performance of 0.30, ranging from 0.03 to 0.57 in the 80% confidence interval. Judge et al.’s research demonstrated that there is a lack of explanation between job satisfaction and performance. They found a more robust connection between job satisfaction and performance in high-complexity jobs (Judge et al., 2000).

In the case of service companies, this paper argues that the quality of working life, represented by HAW, is what determines the quality of the relationship between the salesperson and the customer, and by extension, sales success. HAW has been widely explored and validated in previous research using different samples (Salas-Vallina et al., 2017a; Salas-Vallina and Alegre, 2018a), derived from Fisher’s (2010) conceptualization. It includes job satisfaction, engagement, and affective organizational commitment. These components involve the evaluation of work characteristics (job satisfaction); feelings about the work itself, such as vigor, dedication, and absorption (engagement); and feelings of belonging to the organization (affective organizational commitment). Therefore, HAW is wide enough to overcome the compatibility principle (Harrison et al., 2006), which states that wide attitudinal measures can better predict positive behaviors. For example, in a sample of medical specialists, Salas-Vallina et al. (2017a) found that HAW was positively related to citizenship behavior.

When employees feel happier, they are expected to provide better customer service. The salesperson–customer interaction is always highly important but even more so in service industries, where personal exchanges are crucial to create satisfied customers (Crosby and Stephens, 1987). A service encounter, or “moment of truth” (Normann, 1991), occurs whenever the customer interacts with someone in the company, and on the majority of occasions, this person is the seller. Therefore, the seller is the person who is responsible for the quality of the service. Accordingly, the attitude of the seller will determine whether a sale can be made and whether a good image of the company is created. Consequently, the company must strive to choose good salespeople and make sure they continue to be happy with their job given that their motivation will be transmitted to the customer, thus increasing sales.

A particularly accurate way to measure sales success in banking is cross-selling performance, because it can improve customers’ share of wallet and can add up to 10 times as much value to a company when compared with focusing solely on retention (Coyles and Gokey, 2002). It involves promoting additional products and services to existing customers (Butera, 2000). The important technological and institutional changes in the banking environment have been accompanied by a significant process of concentration, by decreasing interest margins and by a significant increase in income from other sources. As a response to these changes, banks have been reaping efficiency gains, widening the range of products they offer (Allen and Santomero, 2001). In sum, they have increased income by means of cross-selling practices to counter declines in margins. However, at the same time, higher levels of exhaustion and disconnection from work have appeared, thus resulting in a lower quality of life at work.

The JD-R model argues that job demands are physical, psychological, social, and organizational aspects of a job that require a special effort with physiological and/or psychological costs (such as an unfavorable physical or psychological environment). In contrast, job resources refer to the physical, psychological, social, and organizational aspects of a job that help to achieve work objectives, reduce job demands, and stimulate personal growth (Demerouti et al., 2001). The JD-R model suggests that positive attitudes, such as HAW, lead to positive behaviors, such as increased performance. In addition, job resources also improve new and existing job resources, and therefore, HAW could foster service-skill use, thus having a positive impact on cross-selling performance. In a literature review, Bakker and Demerouti (2008) argued that positive attitudes make employees more productive and more willing to go the extra mile. Accordingly, employees’ HAW will positively affect the way they perform, and this will consequently affect their cross-selling skills, although there is no literature on the antecedents of cross-selling. In light of the above, our first hypothesis is:

H1. Happiness at work directly and positively affects cross-selling performance.

#### The Mediating Effect of Service-Skill Use in the Relationship Between HAW and Cross-Selling Performance

It seems that the happy productive worker theory (Judge et al., 2001; Zelenski et al., 2008; Ng et al., 2009; Coo and Salanova, 2018) needs to be further developed, given that predicting performance is complex and depends on different variables. Prior studies have tried to find mediating or moderating variables to better understand the phenomenon. For example, Judge et al. (2001) explained that the moderating effect of job complexity determines the connection between job satisfaction and performance. Korman (1970) presented the self-consistency theory, in which self-esteem moderates the relationship between job satisfaction and performance. Other moderating factors that have been proposed include cognitive ability (Varca and James-Valutis, 1993) and affective disposition (Hochwarter et al., 1999). Interestingly, Wang and Xu (2017) examined how employee skills mediated the relationship between human resource management (HRM) practices and employees’ performance.

Fredrickson (2001) affirmed that positive emotions foster skills and social bonds but also that positive emotions build resources for future performance, predicting long-term productivity. Liao et al. (2010) evidenced the relevance of social exchange in the development of skills. From this point of view, when employees show significant levels of HAW, the effect of mobilizing their resources is higher (Bakker, 2017). Positive attitudes (such as HAW) are fundamental to develop employee skills. For example, engagement has been related to increased civic behavior (Saks, 2019), involving better communication and relationship skills. Happier employees are willing to give their best in an emotional state of passion and involvement, thus better exploiting their skills. Salas-Vallina et al. (2017b) revealed that HAW promotes learning opportunities, and thus the development and use of employees’ skills. Llorens et al. (2007), in a longitudinal study, evidenced that the positive attitude of engagement promoted self-efficacy.

Happiness at work could be an effective motivational mechanism to have a positive impact on service-skill use. The differential aspect of HAW lies in its capacity to energize and invigorate individuals, given that HAW acts as a job resource, thus improving and/or creating new job resources. HAW could make employees more absorbed by their work activities. Positive emotions, such as HAW, can also foster performance by increasing flexibility, creativity, integration, and efficiency of thought (Van de Voorde and Van Veldhoven, 2016). Following the JD-R model, HAW acts as a job resource, in turn fostering positive behaviors (increased service-skill use, namely, communication skills, relationship skills, efficiency, and effectiveness) to deliver superior service and engender customer satisfaction (Wang and Xu, 2017). Compared to other service skill measures, and to the concepts of customer knowledge and expertise, this paper offers a broader concept, and therefore, it is more feasible to relate it to job attitudes (such as HAW) and job behaviors (cross-selling as a consequence). Therefore, HAW could be positively related to service skills.

In addition, this paper suggests that service-skill use might foster cross-selling performance. The skills of empathizing with and relating to customers, together with the ability to provide them with effective and efficient solutions, might make consumers feel more comfortable in the service encounter, attracting higher attention and willingness to buy products and services. In banking, the service encounter relies heavily on interaction, communication skills, relationship skills, and the ability to solve problems in an effective and efficient way that provides service personalization, in which staff serve customers’ needs better. Previous studies have highlighted the lack of research on service-skill use in the service context (Vaerembergh and Holmqvist, 2013). This is surprising, given that good service-skill use is essential in service interactions (Bitner et al., 1990). We argue that service-skill use improves persuasion (Swap et al., 2001) and, therefore, cross-selling performance.

In short, the differentiating factor when two people interact in a business process is the feeling of connection, trust, and attachment derived from service abilities. Thus, employees’ skills enable individuals to improve their outcomes (Obeidat et al., 2016) and performance (Wang and Xu, 2017).

Given all of the above, this paper argues that employees who can provide excellent service through service-skill use will increase cross-selling performance. Accordingly, our second hypothesis is:

H2. Service-skill use mediates the relationship between happiness at work and cross-selling performance.

## Materials and Methods

### Target Population and Sample

Our research used a target population of 3,128 financial service employees from three major banks in Spain, working in frontline banking services in commercial branches focused on business customers. Most of the studies related to positive attitudes are quantitative, in order to connect them to other organizational concepts in a more objective and transposable manner. Our research, which used quantitative methodology, continues with this trend.

The survey was carried out via an online questionnaire, using the Limesurvey software, with the appointment of the human resource department. Items were randomized in order to avoid bias. Participants were asked to provide informed consent before taking part in this research.

The HAW questions were answered by subordinate employees, and the service abilities and cross-selling performance questions were answered by the branch manager. The subordinates were codified, in order to match the responses of both groups. The questionnaire was sent by e-mail in September 2018, explaining its importance and guaranteeing the anonymous treatment of the information furnished. Electronic questionnaires involve cost reduction and the immediate availability of the survey. Two reminders were sent in October and November 2018. A total of 492 valid questionnaires were finally received, from a total sample of 2,417 employees. Branches that had changed their specialization from business customers to retail customers (20.36% response rate) were discarded.

Publishing the results of a survey encourages participants to take part in it (Malhotra et al., 2004). Therefore, all banking employees that accepted taking part in the survey were promised a general report of the study in order to encourage the maximum number of participants.

### Measurement

All the measurement scales used (see Appendix) have been widely validated in previous research.

To measure *HAW*, this paper used Salas-Vallina and Alegre’s (2018a) Likert scale, which comprises nine items, ranging from 1, “totally disagree,” to 7, “totally agree.” Branch subordinates were asked about their level of HAW (i.e., “I would be very happy to spend the rest of my career with this organization”). The principal component analysis showed that the nine items loaded satisfactorily onto one factor. The scale’s α reliability was 0.921.

To measure *service-skill use*, this paper adapted Vaerembergh and Holmqvist’s (2013) and Wang and Xu’s (2017) measurement scales. Branch managers were asked about their subordinates’ service-skill use, in a six-item measurement scale, ranging from 1, “totally disagree,” to 7, “totally agree” (i.e. “My subordinates can easily maintain a good relationship with customers”). The principal component analysis showed that the 10 items loaded satisfactorily onto one factor. The scale’s *α* reliability was 0.929.

*Cross-selling* was measured using Schmitz et al.’s (2014) four-item cross-selling performance scale. Branch managers were asked to estimate, in a range from 1, “totally disagree,” to 7 “totally agree,” the extent to which their subordinates reached the economic cross-buying potential of their customers (i.e., “My subordinates already cater for our customers’ needs for additional products on a broad basis”). The principal component analysis showed that the six items loaded satisfactorily onto one factor. The scale’s α reliability was 0.899.

## Results

### Descriptive Statistics and Psychometric Properties

Table 1 shows descriptive statistics of the sample, in which there were more men than women. They also show that higher educational levels were more frequent in the sample. The average age was 42.2, and the average tenure was 11.4 years. Table 2 shows item loading for each construct.

**TABLE 1:** Gender, educational level, age, and Tenure.

Gender (%)		Education (%)			Age		Tenure	
Men	Woman	Low	Middle	High	*M*	*SD*	*M*	*SD*
54.9	45.1	14.9	37.7	47.4	42.2	9.3	11.4	8.8

**TABLE 2:** Factor loadings of HAW (happiness at work), SKU (service-skill use), and CSP (cross-selling performance).

Factor	Factor loading	Factor	Factor loading	Factor	Factor loading
HAW		Service-skill use		Cross-selling performance	
HAW1	0.81***	SKU1	0.80***	CSP1	0.82***
HAW2	0.76***	SKU2	0.91***	CSP2	0.81***
HAW3	0.84***	SKU3	0.86***	CSP3	0.90***
HAW4	0.91***	SKU4	0.91***	CSP4	0.92***
HAW5	0.90***	SKU5	0.90***		
HAW6	0.82***	SKU6	0.88***		
HAW7	0.73***				
HAW8	0.88***				
HAW9	0.82***				

***p ≤ 0.001.

The psychometric properties of the measurement scales were analyzed following accepted methodology. Dimensionality, content validity, reliability, discriminant validity, and convergent validity were checked.

This paper examined the one-dimensionality of the measurement scales of HAW, service-skill use, and cross-selling performance, by means of confirmatory factor analysis (CFA) using EQS 6.1 software (Table 3). The CFA indicators suggested the one-dimensionality of the HAW construct (*p*-value > 0.05; Bentler-Bonnet Fit Index (BBNFI) = 0.943; Comparative Fit Index (CFI) = 0.992; Root Mean Square Error of Approximation (RMSEA) = 0.067). The evaluation of the service-skill use construct properties also confirmed its one-dimensionality (*p*-value > 0.05; BBNFI = 0.987; CFI = 0.954; RMSEA = 0.053). Once again, the one-dimensionality of cross-selling performance was verified (*p*-value > 0.05; BBNFI = 0.929; CFI = 0.960; RMSEA = 0.059).

**TABLE 3:** Goodness of fit for the one-dimensionality of the measurement scales.

Variable	S-B χ^2^	d.f.	*p*-Value	BBNFI	CFI	RMSEA	NC ( = χ^2^/d.f.)
HAW	23.320	9	0.082	0.923	0.989	0.070	2.591
SA	58.026	27	0.057	0.956	0.970	0.041	2.149
CSP	5.001	2	0.074	0.909	0.936	0.051	2.500

*S-B = Satorra-Bentler; d.f. = degrees of freedom; BBNFI = Bentler-Bonnet Normed Fit Index; CFI = Comparative Fit Index; RMSEA = Root Mean Square Error of Approximation; NC = Normed χ^2^.*

Content validity was confirmed by means of interviews with branch managers and by conducting a literature review.

To evaluate reliability, coefficient alpha and composite reliability were estimated. Table 4 shows that coefficient alphas and composite reliability indicators were acceptable (above 0.70).

**TABLE 4:** Factor correlations, means, standard deviations, composite reliabilities (CRs), average variance extracted (AVE), and Cronbach’s alphas of measurement scales.

	Mean	*SD*	CR	AVE	K	S	HAW	SKU	CSP
1. Happiness at work	4.91	1.33	0.94	0.65	−0.77	−0.81	(0.91)		
2. Service-skill use	5.02	1.19	0.95	0,77	−0.31	0.17	0.39*	(0.88)	
3. Cross-selling performance	4.78	1.68	0.92	0,75	−0.62	-0.44	0.44*	0.33*	(0.90)

* Statistically significant correlation coefficient *p* < 0.01; Cronbach’s alphas are shown on the diagonal. K = kurtosis; S = skewness; s.d. = standard deviation.

Convergent validity assesses whether items in a theoretical model relate to each other. To examine convergent validity, the average variance extracted (AVE) was estimated. Values of AVE above 0.5 indicate convergent validity (Hair et al., 2009). Table 4 shows factor correlations, means, standard deviations, Cronbach’s alphas, composite reliabilities and AVEs. All AVE values are above 0.5, thus showing convergent validity.

We checked discriminant validity for the three scales by comparing two models for each scale. The first model was estimated by constraining the correlation to 1. We proved the discriminant validity of the scales by using pairwise analyses through CFA between all dimensions. Then, we estimated a second model by setting the correlation between these dimensions to unity. The results show that the model fit better when the correlation between dimensions was different from unity, which demonstrates discriminant validity. In addition, the results show that correlation coefficients were significant and below 0.9, which also guaranteed discriminant validity.

Since all variables were gathered from the same source, we checked for common method biases by following Podsakoff et al.’s (2012) method. First, we estimated an Exploratory Factor Analysis (EFA) Harman’s single-factor test. The results indicated that three factors were present (HAW, service-skill use, and cross-selling performance), and the higher covariance explained by one factor was 21.48%, thus confirming that common method bias was not significant. Further, a CFA Harman’s single-factor test, where all the items of the three variables loaded on the same factor, was conducted, showing a poor model fit (χ^2^/degrees of freedom (d.f.) = 5.842; BBNFI = 0.442; CFI = 0.361; RMSEA = 0.248) (Table 6, one-factor model). Second, we also checked a model including the proposed factors and an unmeasured latent factor to control for common method bias (Podsakoff et al., 2012). By adding a latent factor to the CFA model, we connect it to all observed items in the model. Supplementary Graphic 1, which represents the model including the latent factor. We compared the standardized weights from a model without the common latent to the standardized weights of the common latent factor (CLF) model. If differences are lower than 0.200, then common method bias is not a problem. Table 5 shows differences between the full measurement model and the CLF model below 0.2. In addition, Table 6 shows that the additional latent method factor did not significantly improve the model fit [Satorra-Bentler (S-B) χ^2^ = 187.211; d.f. = 130; *p*-value = 0.001; BBNFI = 0.965; CFI = 0.966; RMSEA = 0.040; χ^2^/d.f. = 1.440], compared to the full measurement model (S-B χ^2^ = 212.325; d.f. = 149; *p*-value = 0.001; BBNFI = 0.963; CFI = 0.965; RMSEA = 0.044; χ^2^/d.f. = 1.425). Based on these findings, we can affirm that common method bias did not affect the results of the present research (Podsakoff et al., 2003).

**TABLE 5:** Factor loadings of measurement model, factor loadings of common latent factor (CLF), and difference between loadings of measurement model and common latent factor model.

Construct	Indicator	Factor loading (no CLF)	Factor loading (CLF)	Difference (no CLF - CLF)
**HAW**	HAW1	0.810***	0.808	0.002
	HAW2	0.761***	0.750	0.011
	HAW3	0.845***	0.829	0.016
	HAW4	0.909***	0.881	0.028
	HAW5	0.882***	0.860	0.022
	HAW6	0.823***	0.798	0.025
	HAW7	0.728***	0.721	0.007
	HAW8	0.880***	0.869	0.011
	HAW9	0.824***	0.813	0.011
**Service-skill use**	SKU1	0.803***	0.801	0.002
	SKU2	0.909***	0.904	0.005
	SKU3	0.858***	0.851	0.007
	SKU4	0.911***	0.899	0.012
	SKU5	0.899***	0.880	0.009
	SKU6	0.877***	0.870	0.007
**Cross-selling performance**	CSP1	0.803***	0.778	0.025
	CSP2	0.811***	0.803	0.008
	CSP3	0.904***	0.903	0.001
	CSP4	0.918***	0.909	0.009

**TABLE 6:** One-factor model, full measurement model, and common latent factor model estimation.

Mod.	S-B χ^2^	d.f.	*p*-Value	BBNFI	CFI	RMSEA	NC ( = χ^2^ /d.f.)
One-factor model	887.984	152	0.001	0.442	0.361	0.248	5.842
Full measurement model	212.325	149	0.001	0.963	0.965	0.044	1.425
Common factor model	187.211	130	0.001	0.965	0.966	0.040	1.440

*S-B = Satorra-Bentler; d.f. = degrees of freedom; BBNFI = Bentler-Bonnet Normed Fit Index; CFI = Comparative Fit Index; RMSEA = Root Mean Square Error of Approximation; NC = Normed χ^2^.*

### Analysis of Hypotheses

**FIGURE 1:**
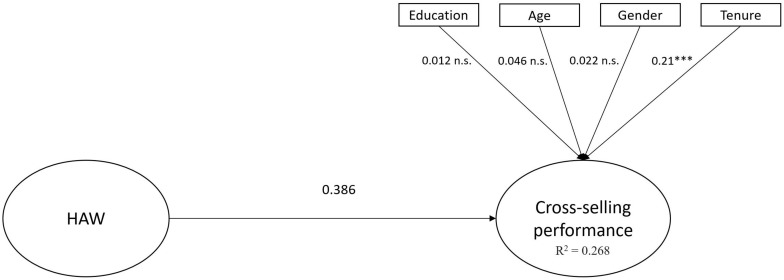
Direct effect model. ^∗∗∗^*p* < 0.001. HAW, happiness at work.

**FIGURE 2:**
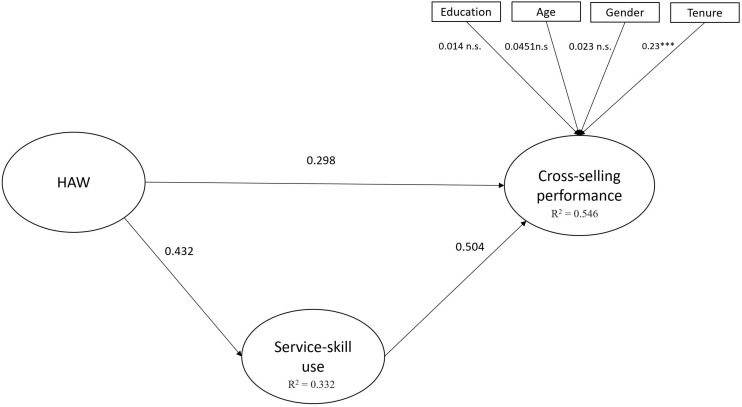
Mediation model. ^∗∗∗^*p* < 0.001.

To evaluate the structural model, we used the coefficient of determination (*R*^2^) of the endogenous latent variables and the strength of the connections between the constructs (Chin, 1998). Bootstrapping was performed to generate standard errors and t-statistics, based on 500 bootstrap samples. Figure 1 shows the results of the direct effect model. Figure 2 shows the results for the mediation model.

The mediation model results reveal that *R*^2^ = 0.546, which means that the theoretical mediation model explained 14.6% of the variance of the construct. This result is higher than 26.8% of the variance explained by the direct effect model. In consequence, we can conclude that the proposed model has a suitable predictive capacity for cross-selling performance. As shown in Figure 1, HAW has a significantly positive relationship with cross-selling performance, thus supporting the first hypothesis. Figure 2 shows a mediating effect of service-skill use in the relationship between HAW and cross-selling performance. In other words, service-skill use significantly contributed to explaining the positive effect of HAW on cross-selling performance. We controlled for age, gender, educational level, and job tenure, following previous research (Groza et al., 2016). Job tenure showed a significant effect on cross-selling performance.

Table 7 shows the results of the effect of HAW on cross-selling performance through service-skill use. First, hypothesis 1 is supported, since HAW is found to have a significant impact on cross-selling performance (β = 0.396, *p* < 0.001). Hypothesis 2 proposes that service-skill use mediates the relationship between HAW and cross-selling performance. In order to check this effect, we examined the total effect of HAW on cross-selling performance and the indirect effect of HAW on cross-selling performance through service-skill use (Preacher and Hayes, 2004). The total effect of HAW on cross-selling performance is significant and different from zero, thus showing a direct relationship between HAW and cross-selling performance. After controlling for service-skill use, the coefficient of the relationship between HAW and cross-selling performance decreases, and the indirect effect through service-skill use is significant and different from zero. Therefore, a mediation effect is observed (Preacher and Hayes, 2004), and hypothesis 2 is supported.

**TABLE 7:** Test results of partial mediation effect: the mediating role of SKU on the relationship between HAW and CSP.

				Percentile	
	Coefficient	*SE*	*t*-Value	Lower	Upper
**Total effect**					
HAW → CSP	0.396***	0.02	58.14		
**Direct effect**					
HAW → CSP	0.314***	0.01	48.36		
HAW → SKU	0.466***	0.02	72.22		
SKU → CSP	0.512***	0.01	98.84		
AGE → CSP	0.074 n.s.	0.02	0.36		
GENDER → CSP	0.026 n.s.	0.03	0.04		
**Indirect effect**					
HAW → SKU → CSP	0.129*	0.01	17.42	0.06	0.14

***p ≤ 0.001, n.s. = non-significant.

## Discussion

Although some studies have shown a significant relationship between positive attitudes (such as job satisfaction) and performance (Ng et al., 2009), other studies have demonstrated a limited correlation in this relationship (Judge et al., 2001). In any case, the literature shows that the happy productive worker relationship is still alive (Coo and Salanova, 2018),

This paper contributes to the literature in different ways. First, it sheds light on the happy productive worker black box by offering a model in which employees experiencing higher levels of well-being improve their performance. In particular, this paper reveals that HAW is a reliable predictor of cross-selling performance. The banking industry is highly demanding and requires exceptional levels of energy and enthusiasm, and this research confirms that employees who show higher passion and energy at work (engagement), who positively evaluate their job conditions (job satisfaction), and who feel involved in the organization (affective organizational commitment) show better cross-selling performance results. This is extremely important, as commercial banks need employees who increase customer loyalty by means of cross-selling. The JD-R model explains these findings, as HAW acts as a job resource that positively contributes to organizational objectives.

Second, past inconsistencies in the happy productive worker literature reveal a lack of explanatory capacity in this relationship. Judge et al.’s (2001) meta-analysis showed the weak relationship between positive attitudes and performance. Performance prediction is complex and depends on different variables, and this might result in problems in the positive attitudes–performance relationship (Judge et al., 2001). In an attempt to solve this lack of consistency, some studies explored how different mediating variables affected the positive attitudes–performance relationship (Hochwarter et al., 1999). Interestingly, the study by Hochwarter et al. (1999) is one of the few works examining the positive attitudes–skills relationship. However, they focused on a narrow positive attitude, namely, job satisfaction. This paper goes one step further and proposes a connection between a wider positive attitude (HAW) and service-skill use, in which the latter mediates the relationship between HAW and cross-selling performance. A central question in the skills literature is the relationship between skills and performance. Results showed that employees who feel happier at work increased their service-skill use. In turn, service-skill use fostered cross-selling performance. Our results reveal that service-skill use improves the effect of HAW on cross-selling performance.

From a managerial perspective, our paper shows that financial services can increase cross-selling performance through the improved use of service skills. It is crucial to understand how customers react when they are served by highly skilled employees. However, scarce research has examined the effect of service-skill use on behavioral outcomes (Vaerembergh and Holmqvist, 2013). Service-skill use has important consequences in the consumption of goods and services (Redondo-Bellón, 1999), and therefore, firms should consider using highly motivated and skilled employees to serve customers.

Salespeople should be able to communicate correctly and need to demonstrate relationship skills, effectiveness, and efficiency. If this does not occur, cross-selling performance results will decrease. Financial services need staff who can cater to their customers’ needs. Firms aim to achieve effective communication between the salesperson and the consumer, and to do this requires the correct demonstration of service skills that offer a sense of security and clarity to the customer, enabling more fluid and precise communication. In addition, service-skill use generates an emotional link between the salesperson and the customer, who will feel more comfortable, recognizing that he or she is receiving personalized treatment. Thus, on the one hand, we suggest that HR departments should focus on employees’ engagement, job satisfaction, and affective organizational commitment by means of HR practices designed to this end. On the other hand, training programs aimed at fostering employees’ service skills should be implemented. All of the above reveals the usefulness and significance of the use of service skills in the sales process.

## Conclusion

In this study, we have attempted to break new ground by mapping a new path in which happier workers are more productive. The study reveals that HAW acts as a job resource that has an effective influence on employees’ performance. In addition, service-skill use was found to play a pivotal role in the relationship between HAW and performance. An important contribution of the study lies in the fact that data were collected from two different sources, which strengthens the reliability of the study and reduces the risk of bias.

The limitations of the paper open up opportunities for future research. First, this research design was cross-sectional. This provides the opportunity to check this model through future two-wave research, which could demonstrate a causal relationship. Second, our data relied on self-reported measures, which involves a risk of bias. Third, our study was limited to the financial services sector. It would be interesting to contrast the importance of service skills in other types of firms. In addition, cross-selling was used as a performance variable, while there are other forms of measuring performance in more subjective ways (Parasuraman et al., 1988), using a combination of objective and subjective methods. In addition, an interesting spiral could emerge in which HAW increases performance, which, in turn, could lead to improved HAW. Hence, future research could explore this gaining spiral through a longitudinal design. In addition, future studies could consider the effect of service-skill use in combination with other personal resources, such as customer orientation, as well as analyze the effect of service-skill use on other constructs, including service recovery performance (see Sommovigo et al., 2019) and organizational citizenship behavior. Finally, our model has focused on the consequences of HAW, yet it has not tackled the antecedents of this concept. There is evidence to support the idea that work circumstances and the interactions between people and their situation have significant implications on HAW (Fisher, 2010). In this vein, future research could test how leader or peer behavior impacts on employees’ HAW. On the one hand, helpful and altruistic behaviors could be beneficial for employees’ HAW (Salas-Vallina and Alegre, 2018b). On the other hand, peers’ self-interest and unethical behaviors are expected to harm HAW. In a recent study, Ruiz-Palomino et al. (2019) found that peers’ unethical behavior increased the negative impact of self-interest (Machiavelianism) on employees’ ethical intentions. They also showed that when peers’ unethical behavior is not present, the negative effect of self-interest on ethical intentions disappears. Hence, future research could test whether the ethicality of people who are not governed by self-interest mindsets could improve HAW.

## Data Availability Statement

The datasets for this manuscript are not publicly available because data belong to a wider dataset shared with other research groups. Requests to access the datasets should be directed to andres.salas@uv.es.

## Ethics Statement

Ethical review and approval was not required for the study on human participants in accordance with the local legislation and institutional requirements. The patients/participants provided their written informed consent to participate in this study.

## Author Contributions

All authors contributed to the data collection and study design. Likewise, all authors drafted the manuscript and worked on several rounds of revision.

## Conflict of Interest

The authors declare that the research was conducted in the absence of any commercial or financial relationships that could be construed as a potential conflict of interest. The reviewer CS declared a past co-authorship with one of the authors AS-V to the handling Editor.

## Appendix

**Happiness at work measurement scale (Salas-Vallina and Alegre, 2018a).**

To which extent do you agree with the following statements, where 1 means “totally disagree,” and 7 “totally agree.”

(1) In my job, I feel strong and vigorous.
(2) I am enthusiastic about my job.
(3) I get carried away when I am working.
(4) How satisfied are you with the nature of the work you perform?
(5) How satisfied are you with the pay you receive for your job?
(6) How satisfied are you with the opportunities which exist in this organization for advancement [promotion]?
(7) I would be very happy to spend the rest of my career with this organization.
(8) I feel emotionally attached to this organization.
(9) I feel a strong sense of belonging to my organization.

**Service-skill use measurement scale, based on Vaerembergh and Holmqvist (2013) and Wang and Xu (2017).**

To which extent do you agree with the following statements, where 1 means “totally disagree,” and 7 “totally agree.”

(1) My subordinates are able to deliver satisfactory services to customers.
(2) My subordinates are well versed in effective ways to provide customers with satisfactory services.
(3) My subordinates are good at solving all kinds of difficulties which customers encounter.
(4) It is very easy for my subordinates to deliver satisfactory services to customers.
(5) My subordinates can easily maintain a good relationship with customers.
(6) My subordinates strive to adapt their language to customers.

**Cross-selling performance measurement scale (Schmitz et al., 2014).**

To which extent do you agree with the following statements, where 1 means “totally disagree,” and 7 “totally agree.”

(1) My subordinates already cater for our customers’ needs for additional products on a broad basis.
(2) Our customers obtain the additional products they require in most cases from my subordinates.
(3) Our customers purchase most of the additional products my subordinates offer them.
(4) My subordinates extensively exploit customers’ potential with regard to additional products.
