# Supplementary figures and images for: Are Happy Workers More Productive? The Mediating Role of Service-Skill Use
Source: Front Psychol. 2020 Mar 27;11:456. doi: 10.3389/fpsyg.2020.00456 (PMC7120033; doi:10.3389/fpsyg.2020.00456)

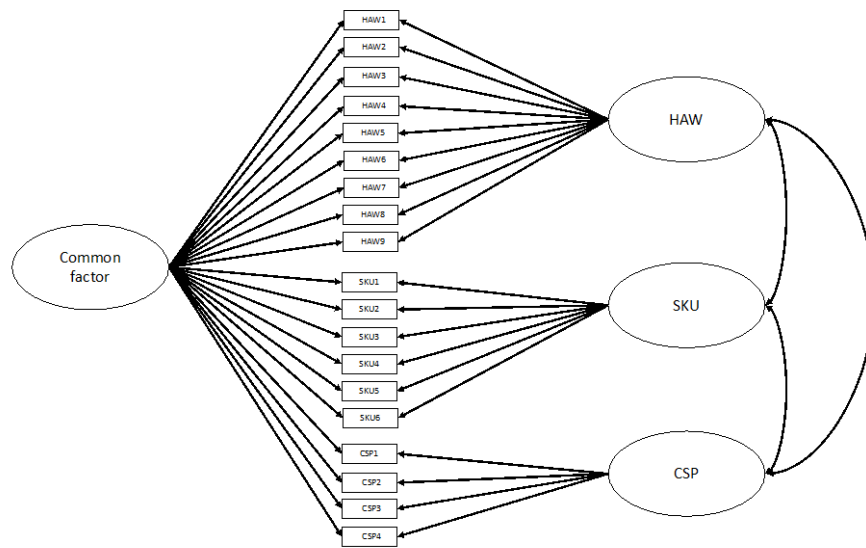

Graphic 1. Supplementary material. Common latent factor model.

Supplement: Supplementary file 1 [file Image_1.PDF]
